# Bilateral Apraxia of Eyelid Closure in Corticobasal Syndrome

**DOI:** 10.1002/alz70857_102357

**Published:** 2025-12-25

**Authors:** Christian Loveland, Estevao Ribeiro, Scott Roye, Claire Delpirou Nouh

**Affiliations:** ^1^ University of Oklahoma Health Sciences Center, Oklahoma City, OK, USA

## Abstract

**Background:**

Eyelid apraxias are relatively uncommon movement disorders, often referring to apraxia of eyelid opening (AEO) while apraxia of eyelid closure (AEC) is even rarer. The later refers to the inability to volitional closure of the eyes while still having normal spontaneous automatic closure. Both are thought related to a dysfunction between neural pathways linking volition and execution of eyelid movement. Corticobasal syndrome (CBS) is a heterogeneous entity, with primary tauopathies including corticobasal degeneration (CBD) being the most frequent underlying pathology, though other etiologies are also possible. AEO is a well‐described clinical feature of CBS; we present a case of CBS with apraxia of eyelid closure (AEC).

**Method:**

Case report

**Result:**

A 72‐year‐old multilingual ambidextrous woman with an history of treated hyperparathyroidism was seen at the neurology clinic with a 5 years history of mood changes, effortful and nonfluent speech progressing to all languages (non‐dominant English most affected), parkinsonism with cogwheel rigidity worse on left while apraxia and dystonic posturing was predominant on the right upper extremity. She had no dysphagia. In addition to above, neurological exam showed apraxia of speech and AEC with preserved reflex blinking, family commenting on preserved eye closure while sleeping. MoCA score was 14/30 and global cognitive impairment was observed on detailed neuropsychological testing. MRI brain showed mild atrophy slightly worse on right as well as bifrontal. DaT‐scan was negative. She deferred other biomarkers testing. Our patient fits possible CBS per Armstrong's criteria. The clinical presentation as well as atrophy pattern favors a 4R‐tauopathy like CBD. A normal DaT scan does not rule it out. AEC is rarer than AEO but can be seen in 4R tauopathies. Some fMRI in AEC research suggested possible insula involvement, an area frequently affected by atrophy in CBD. Other studies on AEC network support dysfunction in the right hemisphere particularly frontoparietal, which are also networks thought involved in emotional expressiveness through gestures and prosody.

**Conclusion:**

AEC is rare, can be seen after ischemic stroke but also in neurodegenerative disorders like primary tauopathies including CBD. More studies are needed to better understand its lesion network and relationship with language ones.

